# Probabilistic prioritization of candidate pathway association with pathway score

**DOI:** 10.1186/s12859-018-2411-z

**Published:** 2018-10-24

**Authors:** Shu-Ju Lin, Tzu-Pin Lu, Qi-You Yu, Chuhsing Kate Hsiao

**Affiliations:** 1 Institute of Epidemiology and Preventive Medicine, College of Public Health, National Taiwan University, Taipei, 10055 Taiwan; 2 Bioinformatics and Biostatistics Core, Center of Genomic Medicine, National Taiwan University, Taipei, 10055 Taiwan

**Keywords:** Association study, Bayesian logistic regression, Competing pathways, Differentially expressed genes, Gene-set analysis, Pathway ranking, Pahtway score

## Abstract

**Background:**

Current methods for gene-set or pathway analysis are usually designed to test the enrichment of a single gene-set. Once the analysis is carried out for each of the sets under study, a list of significant sets can be obtained. However, if one wishes to further prioritize the importance or strength of association of these sets, no such quantitative measure is available. Using the magnitude of *p*-value to rank the pathways may not be appropriate because *p*-value is not a measure for strength of significance. In addition, when testing each pathway, these analyses are often implicitly affected by the number of differentially expressed genes included in the set and/or affected by the dependence among genes.

**Results:**

Here we propose a two-stage procedure to prioritize the pathways/gene-sets. In the first stage we develop a pathway-level measure with three properties. First, it contains all genes (differentially expressed or not) in the same set, and summarizes the collective effect of all genes per sample. Second, this pathway score accounts for the correlation between genes by synchronizing their correlation directions. Third, the score includes a rank transformation to enhance the variation among samples as well as to avoid the influence of extreme heterogeneity among genes. In the second stage, all scores are included simultaneously in a Bayesian logistic regression model which can evaluate the strength of association for each set and rank the sets based on posterior probabilities. Simulations from Gaussian distributions and human microarray data, and a breast cancer study with RNA-Seq are considered for demonstration and comparison with other existing methods.

**Conclusions:**

The proposed summary pathway score provides for each sample an overall evaluation of gene expression in a gene-set. It demonstrates the advantages of including all genes in the set and the synchronization of correlation direction. The simultaneous utilization of all pathway-level scores in a Bayesian model not only offers a probabilistic evaluation and ranking of the pathway association but also presents good accuracy in identifying the top-ranking pathways. The resulting recommendation list of ranked pathways can be a reference for potential target therapy or for future allocation of research resources.

**Electronic supplementary material:**

The online version of this article (10.1186/s12859-018-2411-z) contains supplementary material, which is available to authorized users.

## Background

To evaluate the enrichment of a pathway or gene-set under consideration, several methods for pathway analysis (PA) or gene-set analysis (GSA) have been proposed over the past decades, including the over-representation analysis (ORA), significance analysis of function and expression [1], gene-set enrichment analysis (GSEA) [2], global test [3], and signaling pathway impact analysis (SPIA) [4, 5] (more reviews in [6–8]). The existence of the enrichment of the pathway or gene-set, often a gene ontology term, is sometimes interpreted as the association between the phenotype and the set. A significantly enriched pathway or gene-set would then be recommended for further investigation of subset analysis or target therapy. When several significant pathways are available, these sets may need to be prioritized for future research or for better allocation of limited resources. Two problems arise, however. The first one is that the gene-sets identified by different procedures may not be consistent with each other [6, 9], and the second is the lack of a measure to quantify the strength of association of each set.

The problem in reproducibility can be caused by the discrepancy between the statistical assumptions underlying the approaches and the biological reality of the gene-gene relationship. For instance, genes in the same pathway are often considered independent in several GSAs; while they can correlate with each other because they participate in the same or related biological functions [10–12]. This correlation can inflate type I error rates and reduce power of both univariate and multivariate tests [8, 13, 14]. Another issue of concern is the condition on genes to be included in GSAs. Some analyses including ORA utilize only genes that are differentially expressed (DE), while excluding those exerting mild or weak effect. For instance, ORA uses hypergeometric test for GSA. As pointed out by Rahmatallah and colleagues [9], the power of a gene-set analysis may be influenced by the number of DE genes in that set.

To quantify the strength of association, a common practice is to order the sets based on *p*-values resulting from a certain GSA that is applied to each individual set. Since *p*-value is defined for data more extreme than the observed assuming the null hypothesis is true, its magnitude would not be proper to serve as a quantitative measure for the strength of association [15]; therefore the ranking based on *p*-values would be inappropriate.

To solve these problems, we propose first to summarize the gene expression levels, whether DE or not, in the same pathway with a rank transformation adjusted by direction of correlation. The rank is applied on all samples per gene to depict the relative magnitude of a gene across samples, and the sign of correlation is used to incorporate and synchronize the gene-gene relationship. This procedure is conducted for all gene nodes in the pathway, including those in sub-pathways. We next adopt the Bayesian regression machine to model the degree of association between the pathway and disease status, where the prioritization of competing pathways is carried out based on conditional probabilities. The use of Bayesian model for GSA was considered earlier in [16] for DNA methylation profiling. The rest of the paper is organized as follows. The formulation of the proposed pathway score and the construction of the Bayesian model will be introduced in Section “Methods”. In Section “Results and discussion” we demonstrate the performance of the procedure with simulation studies. The simulated gene expressions are generated either from multivariate Gaussian distributions or from public human expression data to reserve the dependence among genes. The evaluation of performance is based on the type I error rate, percentage of correctly ranking the gene-sets, and the ability to detect the associated pathways. In the same Section we also apply the proposed methodology on a study of high-grade ductal carcinoma in situ with RNA-Seq data and six competing pathways, followed by discussion and conclusion. Note that here we consider a pathway also a gene-set and will interchange the words *pathway* and *gene-set* to refer to a set of genes under investigation.

## Methods

Suppose there are *N* samples and *M* genes or gene nodes in the study. Let *G*_*nm*_ denote the expression value of the *n*-th sample in the *m*-th gene, where *n* = 1, …, *N* and *m* = 1, …, *M*, and let the *N* × *M* matrix **G** contain all expression values, where its column vector is denoted as **G**_·*m*_ = (*G*_1*m*_, *G*_2*m*_, …, *G*_*Nm*_)^*t*^ for the expression of all samples in the *m*-th gene. The rank function is next applied on each gene (column) vector respectively. That is, each column vector in **G** is replaced with the vector **r**(**G**_·*m*_) = (*r*(*G*_1*m*_), *r*(*G*_2*m*_), …, *r*(*G*_*Nm*_))^*t*^.

Next we establish the relationship between genes by first selecting a reference gene, denoting its gene vector as **G**_·*R*_, computing the correlation between this gene and every other column **G**_·*m*_, *m* = 1, …, *M*, in **G**, and recording the direction of the correlation between **G**_·*R*_ and **G**_·*m*_ with the sign function *S*(**G**_·*R*_, **G**_·*m*_). That is, the value of *S*(**G**_·*R*_, **G**_·*m*_) =  *sign* (*corr*(**G**_·*R*_, **G**_·*m*_)) is 1 if they are positively correlated and -1 otherwise. The choice of a fixed reference gene in this procedure is to adjust all correlation directions from the same base unit, i.e., the reference gene in our case, and to avoid cancellation when no base is considered.

### Pathway score

Suppose there are *K* competing pathways, let *C*_*k*_ contain the indices of genes (or gene symbols) in the *k*-th pathway, *k* = 1, …, *K*. If a gene appears in more than one node in the pathway, the frequency of its index is identical to the number of its appearance. Let its cardinality |*C*_*k*_| denote the number of elements in *C*_*k*_. That is, |*C*_*k*_| is the size of the *k*-th pathway. In this pathway, a reference gene is first selected and then a standardized pathway score *p*_*nk*_ is defined to summarize the expression values for the *n*-th sample as

$$ {\displaystyle \begin{array}{l}{p}_{nk}=\frac{Q_{nk}-\sum \limits_{n=1}^N{Q}_{nk}/N}{sd\left({Q}_{1k},\dots, {Q}_{Nk}\right)}\\ {}{Q}_{nk}=\frac{1}{\mid {C}_k\mid}\sum \limits_{m\in {C}_k}r\left({G}_{nm}\right)\times S\left({\mathbf{G}}_{\cdotp R},{\mathbf{G}}_{\cdotp m}\right)\end{array}} $$

Note that *Q*_*nk*_ is the average ranks of the expression levels with signs for the *n*-th sample and *p*_*nk*_ is the standardized score so that the (*p*_*n*1_, *p*_*n*2_, …, *p*_*nK*_) are comparable among the *K* competing pathways. The standard deviation *sd*(*Q*_1*k*_, …, *Q*_*Nk*_) in the denominator is calculated across samples *n* = 1, …, *N* for each fixed *k*. After all pathway scores are computed for each sample, the values are stored in the *N* × *K* matrix **P**.

This proposed pathway score has several advantages. First, the pathway score summarizes for each sample the gene expression through the rank transformation so that the quantity is robust to extreme expression values, as oppose to the direct average. This transformation also standardizes the variability across genes as well as enlarges the heterogeneity. In addition, the product of the function *S* and ranked expression *r*(*G*_*nm*_) in the score integrates all genes by adjusting the direction of correlation between any single gene and the reference. In other words, depending on the direction of the reference gene, the quantity becomes extreme when many genes in the pathway are simultaneously over-expressed or under-expressed. This function *S* can be considered as a synchronizing factor.

### Strength of association and prioritization

To evaluate the strength of association of the *K* pathways, a generalized linear model with a logit link *g* in the case-control setting is employed,

$$ g\left({Y}_n\right)={\beta}_0+\sum \limits_{k=1}^K{\beta}_k{p}_{nk}+{\boldsymbol{\upalpha} \mathbf{X}}_n. $$

The *Y*_*n*_ stands for the disease status of the *n*-th sample, *p*_*nk*_ is the standardized pathway score defined above with the corresponding coefficient *β*_*k*_, and **X**_*n*_ contains other non-genetic explanatory variables associated with this sample.

For the regression coefficient *β*_*k*_, we adopt the maximum posterior probability *P*^(*k*)^ = max {*P*(*β*_*k*_ > 0| **Y**, **X**, **P**), *P*(*β*_*k*_ < 0| **Y**, **X**, **P**)} as a probabilistic evaluation of the strength of association between the *k*-th pathway and the disease status. Here **Y** is the column vector containing disease status of all samples, **X** contains **X**_1_, **X**_2_, …, **X**_*N*_, and **P** is defined as above. The value *P*^(*k*)^ ranges between 0.5 and 1. It represents the degree of association: A value closer to 1 implies a stronger association between the set and the disease status; while a value closer to 0.5 indicates weak or no association. Take two competing pathways *k*_1_ and *k*_2_ for example, if ${P}^{\left({k}_1\right)}$ is larger than ${P}^{\left({k}_2\right)}$, it implies a larger degree of association between *k*_1_ and the disease status than that between *k*_2_ and *Y*. This quantity can now be used to prioritize the *K* competing pathways.

The computation of *P*^(*k*)^ as well as the Markov chain Monte Carlo (MCMC) posterior samples of *β*_*k*_ are carried out with an R package *R2OpenBUGS* to evaluate the posterior probability *P*(*β*_*k*_ > 0| **Y, X, P**) and *P*(*β*_*k*_ < 0| **Y,** **X**, **P**). The code and specification of the full Bayesian model including the distributions of prior and hyper-parameters are provided in Additional files 1 and 2.

## Results and discussion

### Simulation settings

In the following simulation studies, we compare the Bayesian approach with other methods such as GSEA, ORA, global test, frequentist logistic regression with the proposed pathway score (denoted as Logistic (ps)), frequentist logistic regression with the average expression level as the pathway score (Logistic (sum)), and the Fisher’s method. First we generated either 50 or 100 gene expression levels from a multivariate normal distribution with assigned mean, variance, and correlation *ρ* to examine the type I error. The disease status was next determined based on the logistic regression model described above with the intercept *β*_0_ set at 0.01 for a prevalence of 1% and all other regression coefficients set at 0 for no association, or at other given values if association is assumed (described below when data were generated from human genome data for power evaluation). In each replication, 50 cases and 50 controls were generated and the total number of replications is 1000. The value of *ρ* was selected from 0, 0.1, and 0.3 to mimic the independence, weak, and mild correlation among genes in the same set. The *p*-values for the non-Bayesian methods were computed based on asymptotics (if applicable) or 1000 permutations and their significance level was set at 0.05; while for the Bayesian approach, the threshold was set at 0.99 for *P*^(*k*)^ based on 5000 posterior samples. The gene is defined as DE if its single-marker test results in *p* < 0.05.

In addition, we simulated real gene expression data from a large breast cancer study [17] to preserve the relationship among genes. This study contained 13,751 gene expressions from 623 subjects with primary breast cancer. The expression levels were collected from microarray experiments and can be downloaded from Gene Expression Omnibus (GEO) repository (accession number GSE48091). Again, either 50 or 100 genes were randomly selected from this expression data to form a gene-set and to determine the disease status, followed by the analysis with each method to test for association.

### Evaluation of type I error rate

Table 1 lists the type I error rates for each method but SPIA, because SPIA is designed for known pathways and not hypothetical ones. Under the null hypothesis of no pathway association, we note first that the type I error rate does not change much across different values of *ρ*, regardless of the pathway size (50 or 100 genes per set). Second, most tests show reasonable rates: the rates of Logistic (ps), Logistic (sum), GSEA and Fisher’s test are slightly smaller than 0.05, the rates under the Global test are around 0.03 with 50 genes per set and 0.05 with 100 genes, the rates under the Bayesian approach are around 0.02, but that under ORA tends to be as large as 0.1. However, when the data were generated from GSE48091, an inflated type I error rate is apparent for each method, though of different degree. Only the Bayesian regression approach can maintain a rate smaller than 0.05.

**Table 1:** Type I error rates under different settings when the gene-gene correlation ranges from 0 (independence) to mild correlation (*ρ*=0.3), and when data were generated from GSE48091 to preserve the correlation in real application

	50 genes per set				100 genes per set			
	*ρ*=0	*ρ*=0.1	*ρ*=0.3	GSE48091	*ρ*=0	*ρ*=0.1	*ρ*=0.3	GSE48091
Bayesian	0.023	0.022	0.022	0.035	0.024	0.022	0.022	0.025
Logistic (ps)	0.043	0.045	0.046	0.066	0.041	0.040	0.041	0.060
Logistic (sum)	0.040	0.040	0.040	0.160	0.036	0.036	0036	0.041
GSEA	0.047	0.047	0.048	0.069	0.039	0.046	0.045	0.042
Global	0.031	0.030	0.033	0.131	0.047	0.046	0.050	0.159
ORA	0.096	0.094	0.091	0.069	0.057	0.058	0.060	0.085
Fisher’s	0.048	0.048	0.052	0.163	0.049	0.049	0.049	0.201

The size of each set is either 50 or 100. The *p*-values under Global and Fisher’s are derived based on 1000 permutations

### Evaluation of accuracy performance in setting I-V

**Fig. 1:**
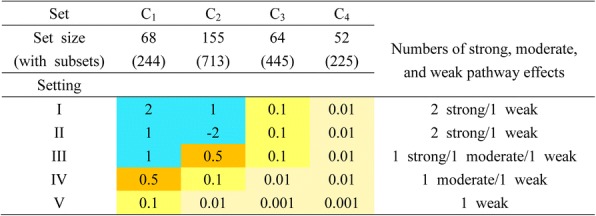
Values of the pathway coefficients in the five simulation settings (I, II, III, IV, and V). The set size is the number of genes in the corresponding set, where the number in parentheses corresponds to genes in the pathway as well as genes in the subsets of the pathway

Next we evaluate the power in terms of the accuracy in selecting the most influential pathway. In other words, the analysis is considered making the correct decision if the *p*-value corresponding to the truly most influential pathway is the smallest; while in the Bayesian approach, the *P*^(*k*)^ has to be the largest. Again we simulated real gene expression data from GSE48091 to preserve the correlation among genes. Furthermore, to compare with SPIA, we deliberately selected p53, Jak-STAT, mTOR, and taste transduction pathways, denoted by *C*_1_, *C*_2_, *C*_3_, and *C*_4_, as the four competing pathways. The contents of these pathways and their sub-pathways all follow the definition in the R package *SPIA*. A pathway with a regression coefficient *β*=1 or 2 is considered exerting strong association, since it corresponds to the odds ratios of log(1) = 2.718 and log(2) = 7.389. Similarly, *β*=0.5, 0.1, 0.01, and 0.001 correspond to 1.649, 1.105, 1.010, and 1.001, and are considered as of moderate, weak, and almost-none association effect. Five simulation settings (I-V) are included and the values of *β* are displayed in Fig. 1.

**Fig. 2:**
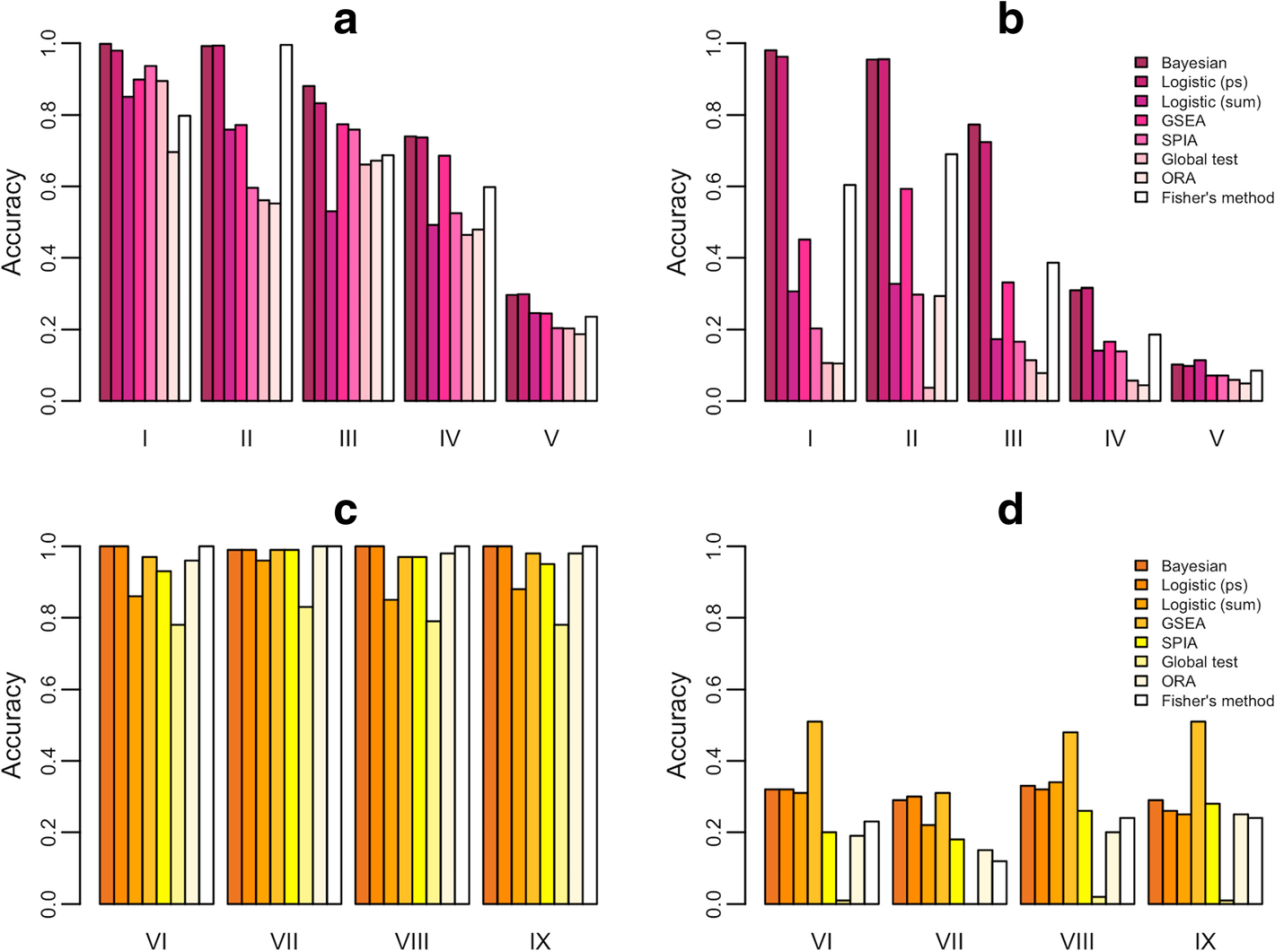
Performance evaluation. **a** The accuracy of selecting the correct top ranking pathway under simulation settings I-V. **b** The accuracy of selecting the correct top two ranking pathways under simulation settings I-V. **c** The accuracy of selecting the correct top ranking pathway under simulation settings VI-IX. **d** The accuracy of selecting the correct top two ranking pathways under simulation settings VI-IX. *P*-values of Global test and Fisher’s method are derived based on asymptotic approximations

Figure 2a demonstrates the accuracy of the 1000 replications in selecting the most influential pathway. For instance, in setting I, the accuracy is defined as the percentage of selecting set *C*_1_ as the top pathway, since *C*_1_ corresponds to the largest absolute regression coefficient 2 in Fig. 1. That is, in each replication, if the *p*-value for *C*_1_ is smaller than *p*-values for *C*_2_ − *C*_4_, respectively, it is counted as accurate for the method. For Bayesian approach, this replication is considered accurate if *P*^(1)^ > *P*^(*k*)^ for all *k* = 2, 3, 4. While in setting II, the accuracy is the percentage that *C*_2_ is selected, since its absolute coefficient 2 is the largest. Among the five settings, generally the Bayesian approach and Logistic (ps) perform the best, except in setting II they are slightly behind the Fisher’s method by only 0.3%. The advantage of the Bayesian approach and Logistic (ps) decreases in setting V; the accuracy of every method is between 20 and 30%. This is under expectation, since all four competing pathways exert weak and similar effects (coefficients between 0.1 and 0.001), making them less differentiable from each other.

The larger accuracy of the Bayesian approach and Logistic (ps) demonstrates the usefulness of the summary measure, the pathway score. In addition, the comparison between Logistic (ps) and Logistic (sum) implies the advantage of incorporating the correlation direction and rank information, which leads to a better performance. Note that here GSEA, SPIA, and Fisher’s method remain in the second best group in identifying the top-ranking pathway. Fisher’s method uses the Chi-square as the asymptotic distribution where significance occurs frequently when the pathway size is large and leads to a large degree of freedom. This may explain why it tends to reach the significant result. The ORA does not perform well because it considers only DE genes and no correlation among genes is included in the analysis.

In Fig. 2b we demonstrate the percentage of correctly selecting the top two ranking pathways. The accuracy evaluation decreases for all methods. Again, the Bayesian approach and Logistic (ps) perform better than the other methods. It appears that these two have the ability to make the correct selection as long as the coefficients are large and separable, such as the first four settings I-IV.

### Evaluation of accuracy performance under single-marker association (setting VI-IX)

In addition to the above pathway-level generation of association, we also consider the scenario of traditional single-marker association, where the gene, instead of the pathway score, is assigned with an effect to associate with the disease. In other words, the disease probability for the *i*-th subject is linked (via a logit scale) to the linear combination $\sum \limits_{j\in {C}_k}{\beta}_{kj}{G}_{ij}$, where *C*_*k*_ contains the index of genes in the associated pathway, *β*_*kj*_ is the effect size of the *j*-th gene in this set, and *G*_*ij*_ is the gene expression of the corresponding gene in this subject. In this scenario, non-zero effect can be assigned to a subset of genes in this set. A randomly selected *M* percent of genes in *C*_1_ are assigned with *n*_1_*β*_1*j*_ = 0.5, *M* percent of genes in *C*_2_ are assigned with *n*_2_*β*_1*j*_ = 1; while the rest 100 − *M* percent in *C*_1_ and *C*_2_, and all genes in *C*_3_ and *C*_4_ are assigned with *n*_*k*_*β*_*kj*_ = 0.01. *n*_*k*_ is the number of causal genes. The four values, 20, 50, 80, and 100, of *M* correspond to the four settings VI, VII, VIII, and IX, respectively.

The performance of accuracy based on 100 replications is displayed in Fig. 2c for selecting the correct top ranking pathway. All methods perform similarly well, where the global test is slightly less powerful (but still around 0.80). In Fig. 2d for selecting the correct top two ranking pathways, all methods perform poorly; the largest power is around 0.50 for GSEA. In other words, no method presents clear advantage.

### Evaluation of power performance for individual pathway

**Fig. 3:**
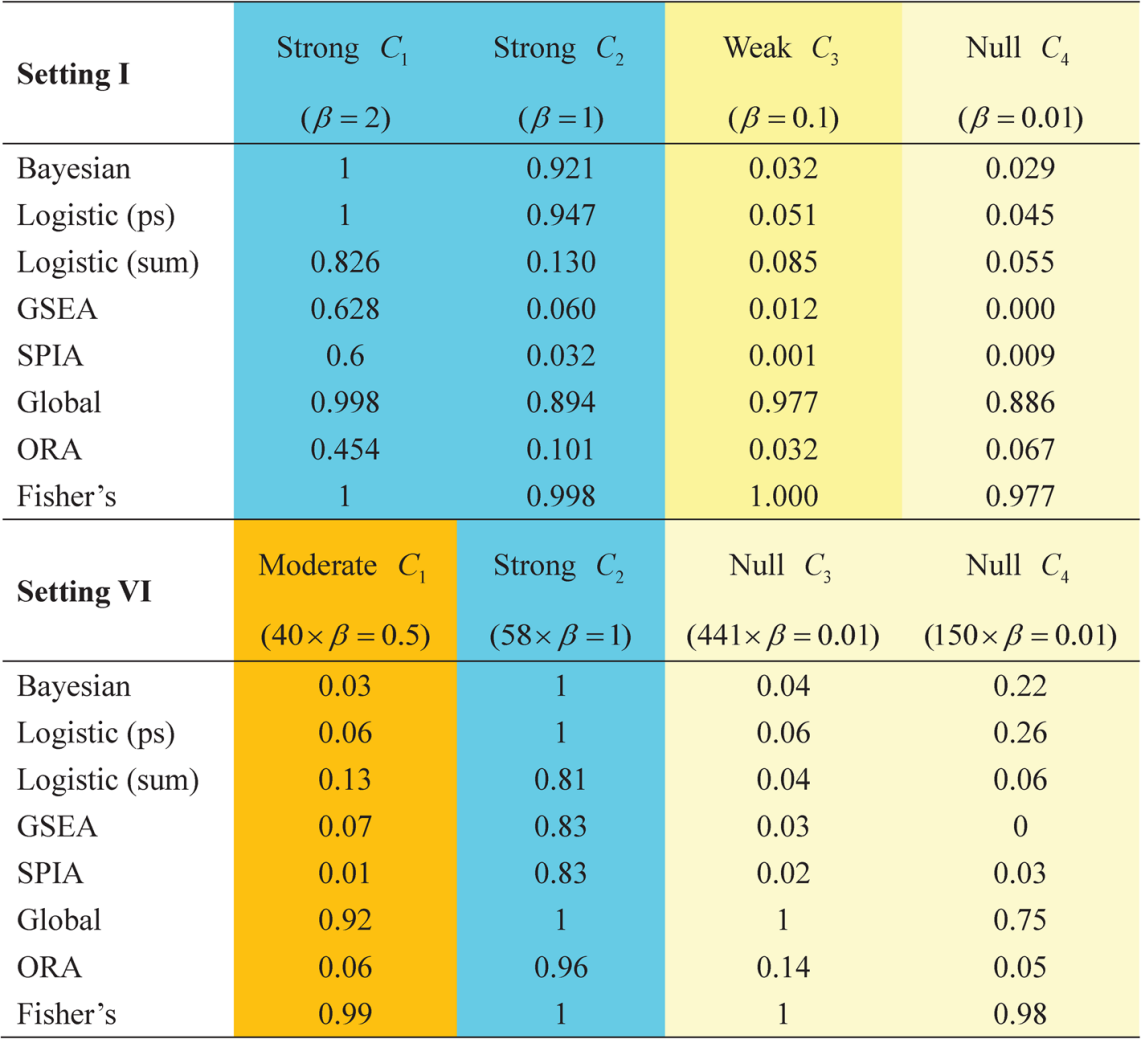
Performance evaluation. The number is the percentage of detected association of each individual gene-set under simulation settings I and VI. *P*-values of Global test and Fisher’s method are derived based on asymptotic approximations

An alternative way to evaluate the performance of these competing methods is to examine their ability to correctly identify each of the associated pathway/gene-set. Using the threshold suggested earlier (i.e., the 0.05 for *p*-value and 0.99 for the maximum posterior probability *P*^(*k*)^), we display in Fig. 3 the percentage of detected association for each pathway. Only settings I and VI are presented here because all show similar patterns. We therefore select I from the first group of simulation design (settings I-V) and VI from the second group (VI-IX).

When the pathway exerts strong association (*C*_1_,*C*_2_ in I and *C*_2_ in VI), four methods (Bayesian, Logistic (ps), Global, and Fisher’s) attain consistently a large power. When the pathway effect is moderate or weak (*C*_3_ in I and *C*_1_ in VI), only Global test and Fisher’s method can detect the association. However, these two methods incorrectly identify, with a high frequency, the pathway with null (almost-none) effects (*C*_4_ in I and *C*_3_,*C*_4_ in VI). In summary, the Bayesian and Logistic (ps) perform similarly well, but the Bayesian approach has a slightly smaller error (*C*_4_ in I and *C*_3_,*C*_4_ in VI).

### Application: High-grade DCIS study

We applied the proposed method to a breast cancer study of pure high-grade ductal carcinoma in situ (DCIS). This study included 25 breast cancer patients and 10 normal controls [18], and applied the next-generation sequencing (NGS) technique to quantify the gene expression. The RNA-Seq data are freely available from National Center for Biotechnology Information (NCBI) GEO database (accession number GSE69240). The data contained read counts from 16,532 genes. Six competing pathways (p53, estrogen, Jak-STAT, mTOR, oocyte meiosis, and taste transduction) were selected specifically for pathway ranking. The first five pathways have been reported to be associated with breast cancer [19]; while the last one was not and is considered here as the null for comparison. The contents of these pathways follow the definition in Kyoto Encyclopedia of Genes and Genomes (KEGG).

**Fig. 4:**
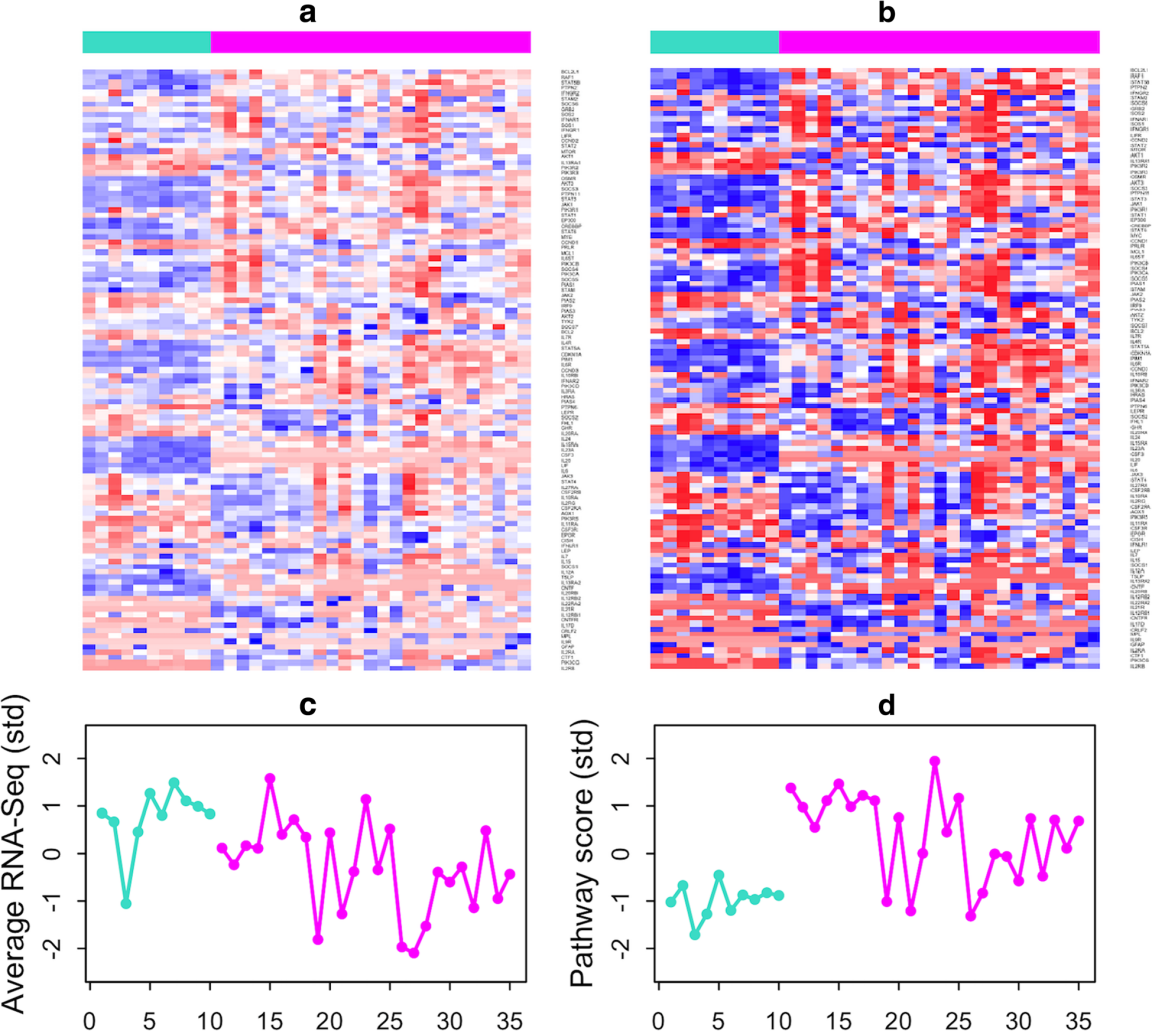
**a** The heatmap of expression counts of genes in the Jak-STAT signaling pathway. **b** The corresponding ranks of expression counts in the same pathway. **c** The summation of expression counts for each sample. **d** The proposed pathway score for each sample

To demonstrate the effect of rank transformation, we display in Fig. 4 the heatmap of gene counts for the Jak-STAT signaling pathway, where Fig. 4a contains the original RNA-Seq data and Fig. 4b includes the ranks of gene counts. The pattern of the relative magnitude does not change, but the contrast in Fig. 4b is much stronger than that in Fig. 4a. In the lower panel, Fig. 4c plots the summation of all gene counts in this pathway for each sample and Fig. 4d shows the value of the proposed pathway score. It can be observed that the 35 summation values in the left tend to overlap with each other; while the pathway scores in the right seem to discriminate better the 10 controls versus the 25 patients.

The six pathways were then investigated under the Bayesian approach, Logistic (ps), Logistic (sum), GSEA, SPIA, ORA, global test, and Fisher’s method, respectively. Table 2 lists either the *P*^(*k*)^ under the Bayesian model, or the *p*-values under other tests. Note that the DE genes here are defined if they pass the single-marker test with Bonferroni correction and the fold-change not between 0.5 and 2. Under each method, the largest and smallest values are in boldface, corresponding to the most influential (top-ranked) and the least influential pathway.

**Table 2:** *P*-values or *P*^(*k*)^ of each pathway under different methods

	p53	estrogen	Jak-STAT	mTOR	oocyte meiosis	taste transduction
Size	68 (290)	99 (838)	158 (1039)	60 (433)	124 (499)	83 (247)
Bayesian	0.717	0.736	**0.895**	0.722	0.611	**0.554**
Logistic (sum)	0.386	**0.447**	0.009	**0.006**	0.038	**0.006**
GSEA	0.264	0.134	0.306	**0.571**	**<1e-4**	0.228
SPIA	**0.195**	0.396	0.266	**0.956**	0.222	0.983
Global	<1e-21	<1e-17	<1e-22	**<1e-23**	<1e-16	**<1e-15**
ORA	**0.036**	0.101	0.275	**0.469**	0.086	0.083
Fisher’s	<1e-214	**0**	<1e-311	<1e-314	**0**	**<1e-67**

Numbers underlined and in boldface indicate the most influential pathway (top-ranked) under each test; while numbers in boldface represent the least influential pathway. The second row lists the number of genes in each pathway, where the number in parentheses includes the genes in sub-pathway. The *p*-values under Global and Fisher’s are asymptotic approximates

For the Bayesian ranking scheme with all six pathways simultaneously included in the same model, Jak-STAT is the most influential because its *P*^(*k*)^ is clearly the largest and around 90%; while the taste transduction is identified the last one with *P*^(*k*)^ close to 50%. For the other tests, the results are contradictory. They can identify one from the first five sets as the most influential, but they may select one from this group as the least important. They are not consistent in identifying the taste transduction pathway as the least important gene-set. GSEA, SPIA and ORA identify mTOR as the least influential pathway; whereas Logistic (sum) recommends mTOR and taste transduction being the most influential. Furthermore, both the global test and Fisher’s method cannot differentiate the six pathways, since the *p*-values are extremely small that it is not meaningful to compare the relative magnitude. The Logistic (ps) cannot be conducted here because the proposed pathway score separate the two groups (healthy vs. disease) almost perfectly, leading to failure in estimating the effect size. For Logistic (sum), the six pathway-specific covariates based on summation of all expression levels cannot be included in the logistic regression model simultaneously due to the small sample size (relative to the number of pathways), therefore the *p*-values in the Table are derived when only one covariate is considered in the analysis.

**Fig. 5:**
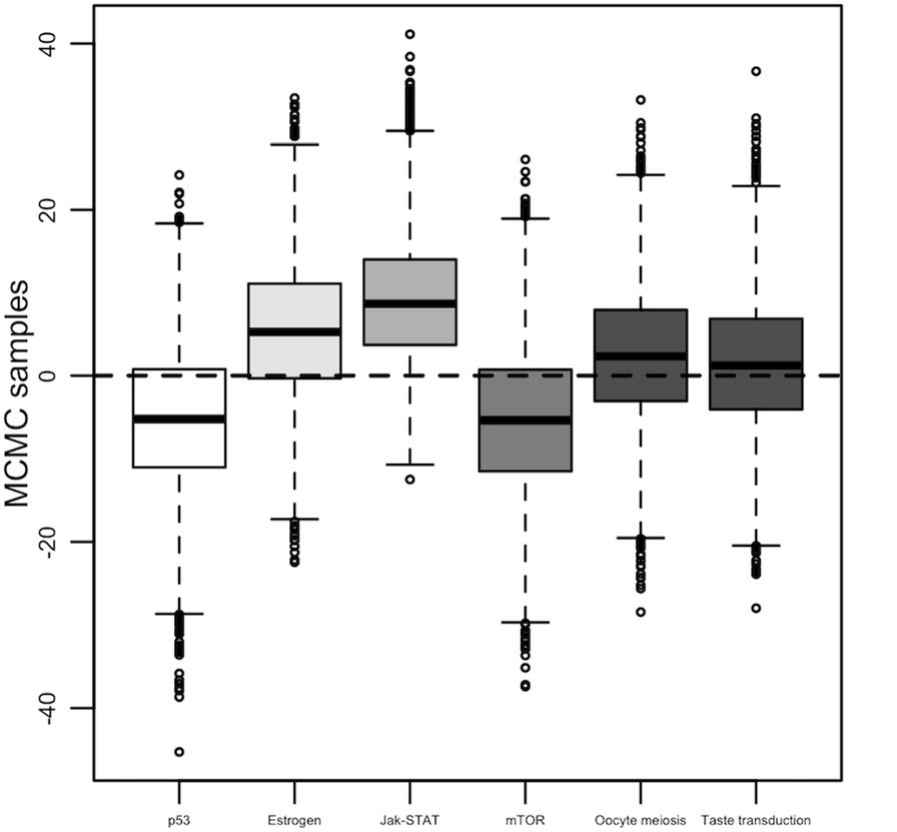
Boxplots of posterior samples of each pathway coefficient under the Bayesian model

The distributions of pathway effect, the regression coefficient, are displayed in Fig. 5 with boxplots of their MCMC posterior samples. The top ranking pathway (the largest *P*^(*k*)^) is Jak-STAT; its pathway score is positively associated with a higher chance of disease risk. This is expected because our proposed score synchronizes directions of all expression counts according to the reference gene, thus follows the same effect direction of the reference. Here *IL17D* is the reference and it does show more expression counts in patients than in controls. This reference is a member of the interleukin 17 family (IL-17) that has been reported to significantly link to tumor progression including the invasive ductal carcinoma, the most common type of breast cancer [20–22]. In other words, a larger value of this summary pathway score implies a higher risk of disease.

The second group of boxplots in Fig. 5 includes three pathways, p53, estrogen, and mTOR, sharing similar values of *P*^(*k*)^ (between 0.72 and 0.74). The degree of association of these three pathways is less significant than that of Jak-STAT, but certainly more substantial than Oocyte meiosis and taste transduction. The least *P*^(*k*)^ is 0.554 for taste transduction, representing a symmetric distribution around zero, which is expected under our hypothesis of no association.

## Conclusions

In this research, we have constructed a measure to summarize the gene-set activity for each subject. This quantity takes into consideration all genes in the same set, accounts for the relationship among genes in terms of correlation direction, and can enhance the contrast of each gene across samples. This measure is then applied in a Bayesian model to evaluate the strength of association between this gene-set and the phenotype of interest, where the posterior probability can represent the degree of association. If multiple gene-sets are of interest, the corresponding probabilities can be ordered to prioritize the gene-sets for future studies. In contrast, other methods consider only one set at a time and use *p*-values for ranking.

There are other advantages in using the Bayesian regression model. The first is its interpretability. The exponent of the regression coefficient is the odds ratio: an odds ratio greater than 1 implies an increase in risk, while a value smaller than 1 implies a decrease. In addition, since the pathway score synchronizes all genes with the reference, the direction of change in pathway risk (i.e., the sign of the regression coefficient) is the same as that of the reference gene. The second advantage is its flexibility. This model can account for quantitative traits when the function *g* is replaced with the identity link, and can be extended to survival analysis and pedigree studies. Furthermore, other covariates like demographic and environmental variables can be included to account for other effects.

For the choice of the reference gene in the gene-set, our criteria include (1) the hub gene, with many neighboring genes being either its up- or down-regulating entities; (2) the one acting as an upstream gene in the set, particularly in a signaling pathway; and (3) the gene with a large variation in expression level. For the breast cancer NGS study, we deliberately selected *TP53*, *ADCY1*, *IL17D*, *TNF*, *CALML5*, and *TAS1R3* as the reference for p53, Estrogen, Jak-STAT, mTOR, oocyte meiosis, and taste transduction pathways, respectively. Following the criteria, these genes are all connected to many nodes in the same pathway, locate in the upstream, and show greater variation then others. There may be more than one such gene in each set. For example, for the Jak-STAT pathway, we have tried *LEP*, *CNTFR*, *GHR*, *IIL7R*, *IL20RB*, and *IL23A* and the results all support the same top ranking pathway. Table 3 lists the corresponding probability *P*^(*k*)^ when these genes are used as the reference, as well as the posterior mean for the regression coefficient. Notice that the sign of the mean is the same as that of the reference gene, i.e., it is positive if the gene is over-expressed in the diseased group and negative if under-expressed. A systematic investigation would be necessary to find an optimal choice.

**Table 3:** Probability *P*^(*k*)^, mean of the regression coefficient *β*, and if over- or under-expressed in the diseased group when different reference gene is considered in the JAK-STAT pathway for the breast NGS study

Reference Gene	*LEP*	*CNTFR*	*GHR*	*IL7R*	*IL20RB*	*IL23A*
Gene symbol	3952	1271	2690	3575	53,833	51,561
*P* ^( *k*)^	0.889	0.886	0.880	0.873	0.844	0.812
Mean	9.05	9.03	8.54	6.71	−8.23	−7.68
Over/under	over	over	over	over	under	under

**Fig. 6:**
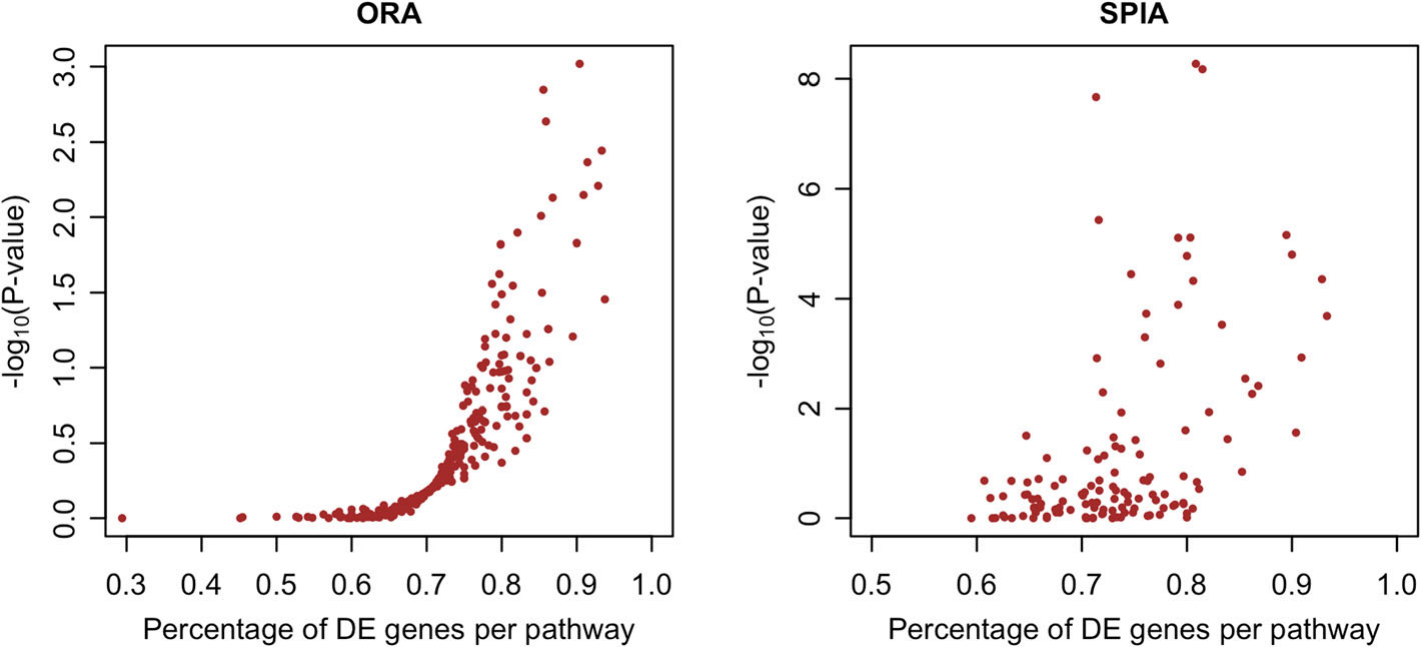
The relationship between the percentage of DE genes and the negative base 10 logarithm of *p*-value under ORA (left, for 289 pathways in KEGG) and SPIA (right, for 130 signaling pathways in KEGG) test, respectively. The linear correlation is 0.80 in the left and 0.49 in the right

Several issues are worth noting here. First, when the purpose is to screen a large collection of gene-sets and not to prioritize a limited set of candidate pathways, the current Bayesian model may not be able to accommodate all sets in a single model, especially when the sample size is not large enough to support statistical inference. In this case, a pre-selection procedure is advised. One may adopt the proposed pathway score as a pre-selection tool in a frequentist logistic regression model for binary response outcomes or in the usual linear regression for quantitative response variables. Based on the performance of single-pathway test in the simulations, although Logistic (ps) has the tendency to provide false positive results, it is easy to use with large power. The resulting sets can next enter the Bayesian procedure for prioritization. The use of other tests like ORA and SPIA in the pre-selection stage would need special attention in the number of DE genes. The set tends to be significant if the number is large. This relationship is demonstrated in Fig. 6 for the breast cancer NGS study. The *p*-value from such single-pathway test, however, cannot replace the ranking procedure. Inference based on a joint model, such as the one proposed in this research, would be preferred.

Second, our proposed procedure is in spirit closer to a self-contained than a competitive test, when adopting the definition in Goeman and Buhlmann [10]. This is because the problem we are dealing with is the association strength of gene-sets. In other words, only sets included in the analysis are under investigation. These sets need not to compete with genes outside the sets. On the other hand, our procedure goes beyond a self-contained test, because we are trying to evaluate the degree of association, not just to test if the association exists. The future aim is to enlarge the list of candidate pathways when all, not just candidates, are included for exploratory and screening purposes.

## Additional files

Additional file 1: R code for computing the pathway score and conducting Bayesian analysis. (TXT 9 kb)

Additional file 2: the R document for the analysis of two hypothetical examples. (PDF 214 kb)

## Abbreviations

DCIS: Ductal carcinoma in situ
DE: Differentially expressed
GEO: Gene expression omnibus
GSA: Gene-set analysis
GSEA: Gene-set enrichment analysis
KEGG: Kyoto Encyclopedia of Genes and Genomes
MCMC: Markov chain Monte Carlo
NCBI: National Center for Biotechnology Information
NGS: Next-generation sequencing
ORA: Over-representation analysis
PA: Pathway analysis
RNA-Seq: RNA-sequencing
SPIA: Signaling pathway impact analysis
