# Supplementary material for: Probabilistic prioritization of candidate pathway association with pathway score
Source: BMC Bioinformatics. 2018 Oct 24;19:391. doi: 10.1186/s12859-018-2411-z (PMC6201593; doi:10.1186/s12859-018-2411-z)
Supplement: Supplementary file 2 — the R document for the analysis of two hypothetical examples. (PDF 214 kb) [file 12859_2018_2411_MOESM2_ESM.pdf]

Example

This document contains two functions, stand.path.score and Bayes.model. The first one computes the standardized pathway score for any given pathway, and the second function performs Bayesian logistic regression for 1 or more than 1 pathways.

Two examples are provided in this document. Input data and example output are attached. The gene expression data for illustration were a subset of the data with accession number GSE69240 from NCBI GEO database.

Note: For the Bayesian analysis, "OpenBUGS" and the R packages "R2OpenBIGS" need to be installed.

```
install.packages("R2OpenBUGS")
library(R2OpenBUGS)
```

Read "exp__data.csv" into R. The file "exp__data" contains information about genes and gene expression data.

```
exp_data <- read.csv("exp_data.csv")
head(exp_data, 10)
##    EntrezGeneID  GeneID GSM1695870 GSM1695872 GSM1695873 GSM1695874 GSM1695875
## 1             1  gene_1  0.9088444   1.044057   1.942943   1.691947   1.322304
## 2             2  gene_2  7.5292076   8.444080   4.811552   5.558894   9.208396
## 3             3  gene_3  1.9527386   2.208967   2.906584   1.903236   2.270081
## 4             4  gene_4 10.5340344   9.984337  10.637549  10.120771  12.168273
## 5             5  gene_5  6.3634338   5.857818   6.909188   6.328964   6.369530
## 6             6  gene_6  8.4258386   8.368376   8.156956   8.198807   8.546982
## 7             7  gene_7  3.0020773   3.556041   4.165459   3.379569   4.023173
## 8             8  gene_8  6.2478619   4.906205   4.017458   4.858359   6.346748
## 9             9  gene_9  3.6741631   4.281410   4.713096   3.830817   4.276942
## 10           10 gene_10  4.2825610   4.645635   3.922734   4.419960   4.379581
##    GSM1695880 GSM1695891 GSM1695898 GSM1695899 GSM1695900
## 1    1.502372   4.623528   2.283847   3.922753   1.546626
## 2   10.178030   8.834743   8.590437   7.755531  10.068061
## 3    3.122389   4.774500   2.441035   2.672881   5.217039
## 4    7.636224   7.723282   7.836271   4.918539   6.791421
## 5    5.760459   5.500309   6.386785   5.729244   5.676942
## 6    6.836560   6.283488   6.749332   6.346691   6.626439
## 7    3.560224   4.396000   4.682381   4.679283   4.386537
## 8    5.419593   4.868473   5.038480   4.961271   7.507087
## 9    5.406525   5.648951   5.109098   5.574989   4.321809
## 10   3.844894   5.581838   4.627626   4.618284   4.487500
```

Example 1: Bayesian analysis with two competing pathways A and B

Step 1. Read "path_A.csv" into R. "path_A" is a pathway including 20 genes, as shown in the following network plot.

```
path_A <- read.csv("path_A.csv")
dim(path_A); head(path_A, 10)
## [1] 20  2
##    geneid   symbol
## 1     182 gene_182
## 2     202 gene_202
## 3     262 gene_262
## 4     301 gene_301
## 5     588 gene_588
## 6     318 gene_318
## 7     327 gene_327
## 8     350 gene_350
## 9     478 gene_478
## 10    482 gene_482
```

Note: if there exists gene symbols not in the "exp_data.csv", they will be excluded in the following analysis.

Network plot of "path_A"

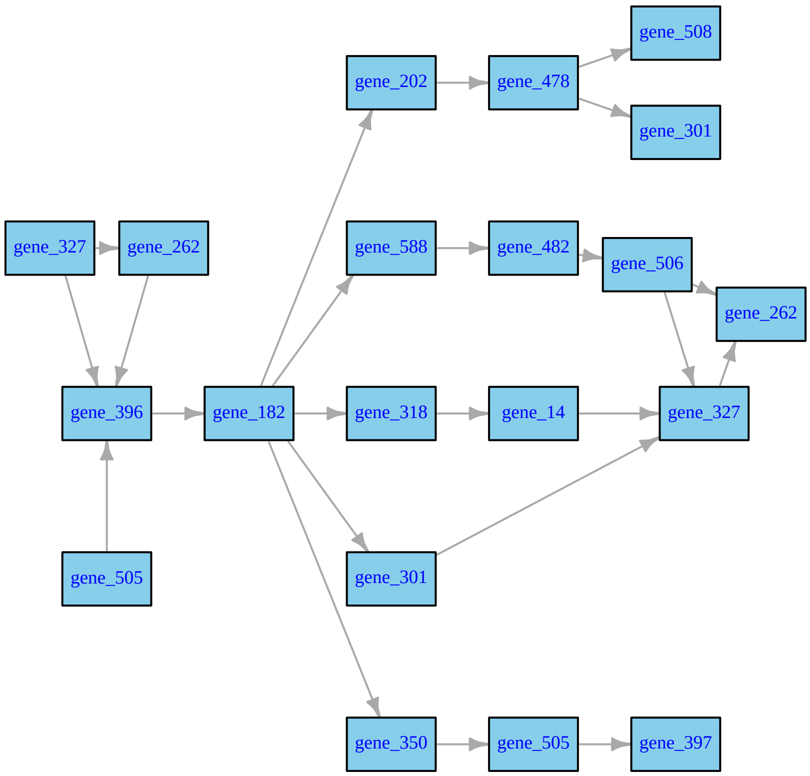

Step 2. Read "path_B.csv" into R. "path_B" is a pathway including 16 genes, as shown in the following network plot.

```
path_B <- read.csv("path_B.csv")
dim(path_B); head(path_B, 10)
## [1] 16  2
##    geneid   symbol
## 1     589 gene_589
## 2      61  gene_61
## 3      61  gene_61
## 4      62  gene_62
## 5      63  gene_63
## 6      63  gene_63
## 7      65  gene_65
## 8      66  gene_66
## 9     590 gene_590
## 10    590 gene_590
```

Note: if there exists gene symbols not in the "exp_data.csv", they will be excluded in the following analysis.

Network plot of "path_B"

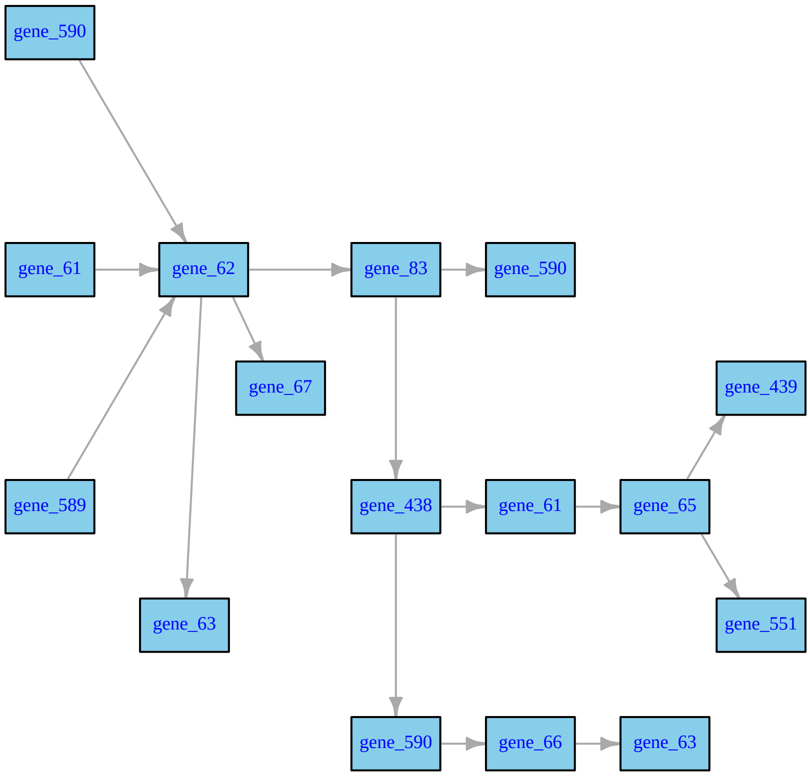

Step 3. Execute "Bayes.model" function. The reference genes in the option "ref.gene.ID" are chosen following the requirements stated in the paper.

```
path_data_AB <- list(path_A, path_B)
result_AB <- Bayes.model(exp.data=exp_data, status=c(rep(0,5),rep(1,5)),
                    pathway.name=c("path_A","path_B"), pathway.data=path_data_AB,
                    no.path=2, ref.gene.ID=c(396, 62), exp.ID="EntrezGeneID",
                    path.ID="geneid", model.file="C:/mymodel_1.odc", no.iter=6000,
                    no.chains=1, no.burnin=1000, no.thin=10)
```

Step 4. Show the results.

```
names(result_AB)
## [1] "rank.table"          "summary"             "infor"
## [4] "posterior.sample"    "history"             "stand.pathway.score"
result_AB$rank.table
##   Pathway Rank Posterior probability
## 1  path_A    1                0.9822
## 2  path_B    2                0.8734
result_AB$summary
##                 mean       sd          2.5%          25%      50%      75%
## beta0     1.5977669 5.532562 -9.074200e+00 -2.036250000  1.42000  4.99275
## beta1[1] 10.9669887 6.533387  5.326300e-01  6.040250000 10.27000 15.05250
## beta1[2]  7.8660324 7.044785 -4.625050e+00  2.806500000  7.36900 12.36000
## deviance  0.4091955 1.025052  6.339725e-08  0.000632575  0.02206  0.28840
##             97.5%
## beta0    13.03075
## beta1[1] 25.27200
## beta1[2] 22.86000
## deviance  3.52030
result_AB$infor
##       pD    DIC Sample size
## 1 0.4092 0.8183          10
head(result_AB$posterior.sample, 1)
##       beta0 beta1[1] beta1[2] deviance
## [1,] 0.3623    3.323    19.19   0.2766
head(result_AB$history, 1)
##      beta0 beta1[1] beta1[2] deviance
## [1,] 7.028    23.55    6.028  2.3e-06
head(result_AB$stand.path.score, 1)
## NULL
```

Example 2: Bayesian analysis with two competing pathways C and D

Step 1. Read "path_C1.csv" into R. "path_C1" is a pathway including 10 genes and two subpathways called "path_C2" and "path_C3".

```
path_C1 <- read.csv("path_C1.csv")
dim(path_C1); head(path_C1, 10)
## [1] 10  2
##    geneid   symbol
## 1     182 gene_182
## 2     202 gene_202
## 3     262 gene_262
## 4     301 gene_301
## 5     588 gene_588
## 6     318 gene_318
## 7     327 gene_327
## 8     350 gene_350
## 9     478 gene_478
## 10    482 gene_482
```

Note: if there exists gene symbols not in the "exp_data.csv", they will be excluded in the following analysis.

Network plot of "path_C1"

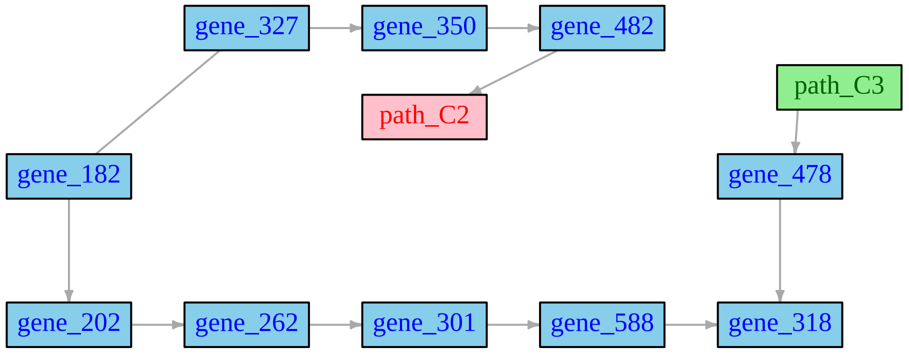

Step 2. Read "path_C2.csv" into R. "path_C2" is a subpathway of "path_C1" and it includes 10 genes.

```
path_C2 <- read.csv("path_C2.csv")
dim(path_C2); head(path_C2, 10)
## [1] 10  2
##    geneid   symbol
## 1      14  gene_14
## 2     262 gene_262
## 3     301 gene_301
## 4     327 gene_327
## 5     396 gene_396
## 6     397 gene_397
## 7     505 gene_505
## 8     505 gene_505
## 9     506 gene_506
## 10    508 gene_508
```

Note: if there exists gene symbols not in the "exp_data.csv", they will be excluded in the following analysis.

Network plot of "path_C2"

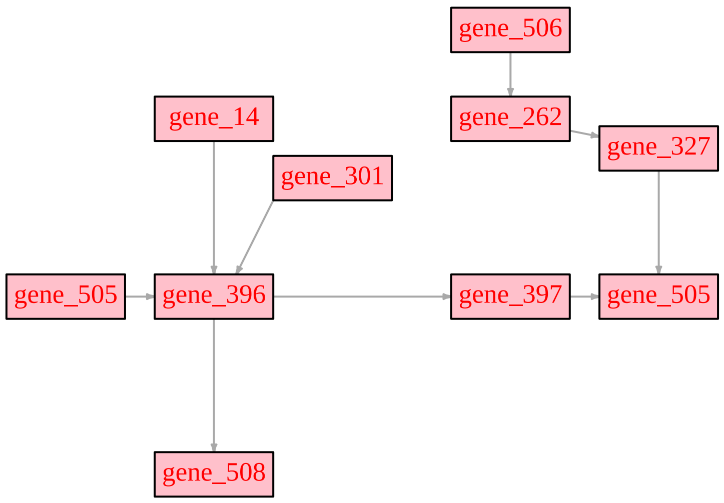

Step 3. Read "path_C3.csv" into R. "path_C3" is a subpathway of "path_C1" and it includes 10 genes.

```
path_C3 <- read.csv("path_C3.csv")
dim(path_C3); head(path_C3, 10)
## [1] 10  2
##    geneid   symbol
## 1      41  gene_41
## 2      42  gene_42
## 3      87  gene_87
## 4      88  gene_88
## 5     120 gene_120
## 6     121 gene_121
## 7     182 gene_182
## 8     182 gene_182
## 9     191 gene_191
## 10    191 gene_191
```

Note: if there exists gene symbols not in the "exp_data.csv", they will be excluded in the following analysis.

Network plot of "path_C3"

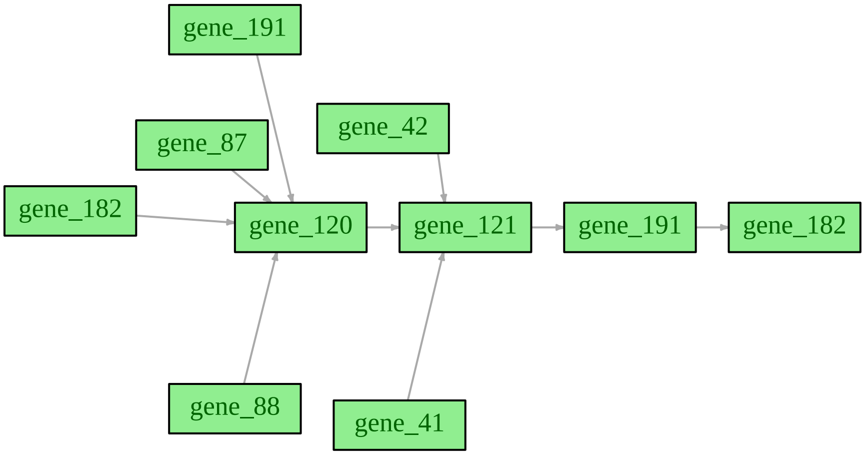

Step 4. Read "path__D1.csv" into R. "path__D1" is a pathway and it includes 6 genes and a subpathway called "path__D2".

```
path_D1 <- read.csv("path_D1.csv")
dim(path_D1); path_D1
## [1] 6 2
##   geneid   symbol
## 1    589 gene_589
## 2     65  gene_65
## 3    590 gene_590
## 4    438 gene_438
## 5    439 gene_439
## 6    551 gene_551
```

Note: if there exists gene symbols not in the "exp__data.csv", they will be excluded in the following analysis.

Network plot of "path__D1"

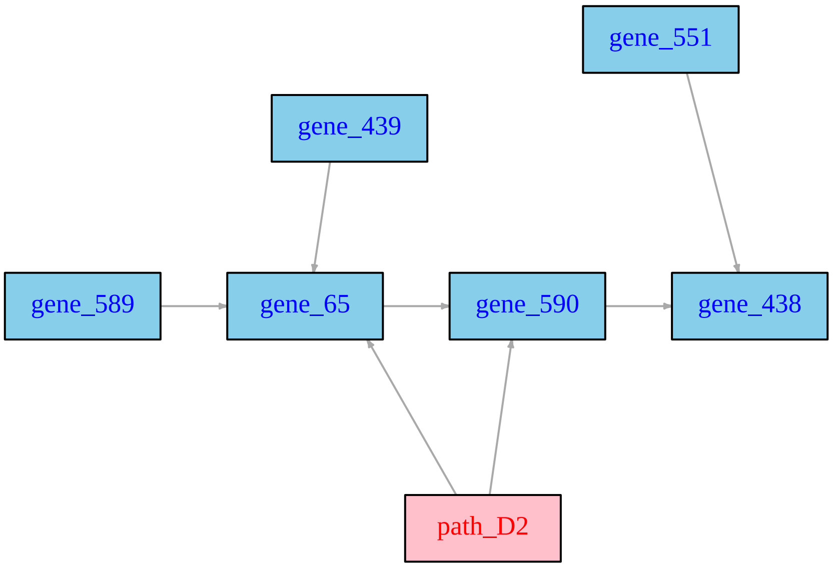

Step 5. Read "path_D2.csv" into R. "path_D2" is a subpathway of "path_D1" and it includes 10 genes.

```
path_D2 <- read.csv("path_D2.csv")
dim(path_D2); path_D2
## [1] 10  2
##    geneid   symbol
## 1      61  gene_61
## 2      61  gene_61
## 3      62  gene_62
## 4      63  gene_63
## 5      63  gene_63
## 6      66  gene_66
## 7     590 gene_590
## 8     590 gene_590
## 9      67  gene_67
## 10     83  gene_83
```

Note: if there exists gene symbols not in the "exp_data.csv", they will be excluded in the following analysis.

Network plot of "path_D2"

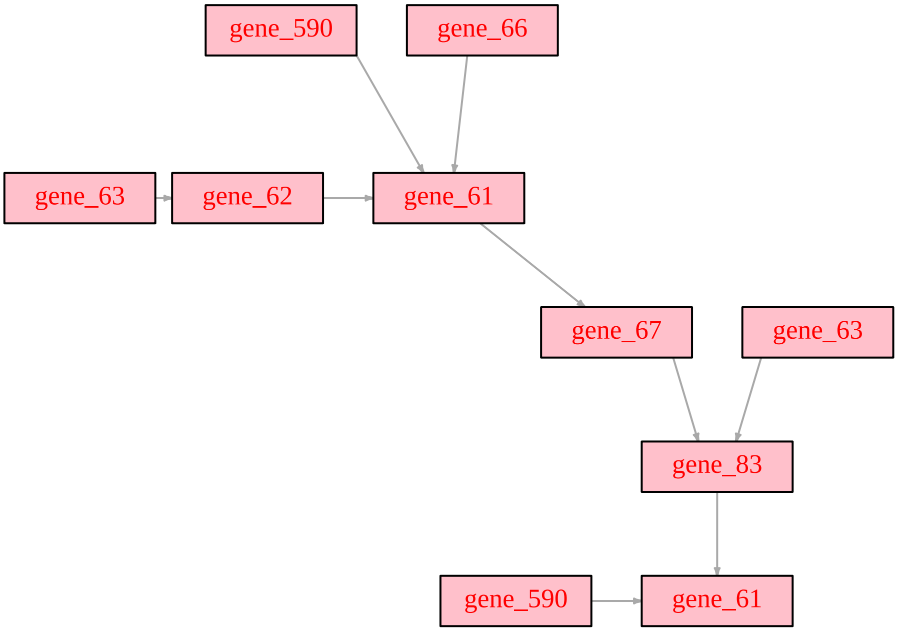

Step 6. Merge the subpathways and execute "Bayes.model" function. The reference genes in the option "ref.gene.ID" are chosen following the requirements stated in the paper.

```
path_data_CD <- list(rbind(path_C1, path_C2, path_C3), rbind(path_D1, path_D2))
result_CD <- Bayes.model(exp.data=exp_data, status=c(rep(0,5),rep(1,5)),
                         pathway.name=c("path_C", "path_D"), pathway.data=path_data_CD,
                         no.path=2, ref.gene.ID=c(397, 61), exp.ID="EntrezGeneID",
                         path.ID="geneid", model.file="C:/mymodel_2.odc", no.iter=6000,
                         no.chains=1, no.burnin=1000, no.thin=10)
```

Step 7. Show the results.

```
names(result_CD)
## [1] "rank.table"         "summary"              "infor"
## [4] "posterior.sample"    "history"              "stand.pathway.score"
result_CD$rank.table
##   Pathway Rank Posterior probability
## 2  path_D    1                     1
## 1  path_C    2                0.8896
result_CD$summary
##                 mean       sd         2.5%        25%      50%      75%
## beta0     2.9990076 3.498239 -2.980475000  0.5572500  2.70950  5.12650
## beta1[1]  5.3244326 4.584771 -2.320500000  2.0342500  4.86050  8.10075
## beta1[2] 14.4529882 5.742287  4.940499011 10.3474991 13.88000 18.00000
## deviance  0.9728498 1.499480  0.001807623  0.0737175  0.35185  1.21825
##               97.5%
## beta0    10.700750
## beta1[1] 15.480250
## beta1[2] 26.954237
## deviance  5.398025
result_CD$infor
##      pD   DIC Sample size
## 1 0.944 1.917          10
head(result_CD$posterior.sample, 1)
##      beta0 beta1[1] beta1[2] deviance
## [1,] 6.733     8.49     16.3  0.06977
head(result_CD$history, 1)
##       beta0 beta1[1] beta1[2] deviance
## [1,] -3.652    4.757    10.47    4.569
head(result_CD$stand.path.score, 1)
## NULL
```
